# The genetics of gene expression in a *Caenorhabditis elegans* multiparental recombinant inbred line population

**DOI:** 10.1093/g3journal/jkab258

**Published:** 2021-08-10

**Authors:** Basten L Snoek, Mark G Sterken, Harm Nijveen, Rita J M Volkers, Joost Riksen, Philip C Rosenstiel, Hinrich Schulenburg, Jan E Kammenga

**Affiliations:** Laboratory of Nematology, Wageningen University, NL-6708 PB Wageningen, The Netherlands; Theoretical Biology and Bioinformatics, Utrecht University, 3584 CH Utrecht, The Netherlands; Laboratory of Nematology, Wageningen University, NL-6708 PB Wageningen, The Netherlands; Bioinformatics Group, Wageningen University, NL-6708 PB Wageningen, The Netherlands; Laboratory of Nematology, Wageningen University, NL-6708 PB Wageningen, The Netherlands; Laboratory of Nematology, Wageningen University, NL-6708 PB Wageningen, The Netherlands; Institute for Clinical Molecular Biology, University of Kiel, 24098 Kiel, Germany; Competence Centre for Genomic Analysis (CCGA) Kiel, University of Kiel, 24098 Kiel, Germany; Zoological Institute, University of Kiel, 24098 Kiel, Germany; Max Planck Institute for Evolutionary Biology, 24306 Ploen, Germany; Laboratory of Nematology, Wageningen University, NL-6708 PB Wageningen, The Netherlands

**Keywords:** multiparental RILs, expression QTL, eQTL, SNPs, *C. elegans*, MPP, Multiparental Populations, Multiparent Advanced Generation Inter-Cross (MAGIC)

## Abstract

Studying genetic variation of gene expression provides a powerful way to unravel the molecular components underlying complex traits. Expression quantitative trait locus (eQTL) studies have been performed in several different model species, yet most of these linkage studies have been based on the genetic segregation of two parental alleles. Recently, we developed a multiparental segregating population of 200 recombinant inbred lines (mpRILs) derived from four wild isolates (JU1511, JU1926, JU1931, and JU1941) in the nematode *Caenorhabditis elegans*. We used RNA-seq to investigate how multiple alleles affect gene expression in these mpRILs. We found 1789 genes differentially expressed between the parental lines. Transgression, expression beyond any of the parental lines in the mpRILs, was found for 7896 genes. For expression QTL mapping almost 9000 SNPs were available. By combining these SNPs and the RNA-seq profiles of the mpRILs, we detected almost 6800 eQTLs. Most *trans*-eQTLs (63%) co-locate in six newly identified *trans*-bands. The *trans*-eQTLs found in previous two-parental allele eQTL experiments and this study showed some overlap (17.5–46.8%), highlighting on the one hand that a large group of genes is affected by polymorphic regulators across populations and conditions, on the other hand, it shows that the mpRIL population allows identification of novel gene expression regulatory loci. Taken together, the analysis of our mpRIL population provides a more refined insight into *C. elegans* complex trait genetics and eQTLs in general, as well as a starting point to further test and develop advanced statistical models for detection of multiallelic eQTLs and systems genetics studying the genotype–phenotype relationship.

## Introduction

Investigation of the genotype–phenotype relationship is at the heart of genetic research. The detection and description of allelic variants and genetic mechanisms have been a demanding task due to the quantitative nature of most phenotypic variation. Quantitative trait locus (QTL) mapping has been one of the methods of choice for finding the loci on which these allelic variants can be found. Many functional polymorphisms in plants and animals, including many model species such as model nematode *Caenorhabditis* *elegans*, have been discovered using QTL mapping (Tijsterman *et al.* 2002; Rogers *et al.* 2006; Kammenga *et al.* 2007; Gloria-Soria and Azevedo 2008; Palopoli *et al.* 2008; Reiner *et al.* 2008; Seidel *et al.* 2008; McGrath *et al.* 2009; Reddy *et al.* 2009; Bendesky *et al.* 2011; Seidel *et al.* 2011; Bendesky *et al.* 2012; Ghosh *et al.* 2012; Andersen *et al.* 2014; Noble *et al.* 2015; Schmid *et al.* 2015; Cook *et al.* 2016; Greene *et al.* 2016; Large *et al.* 2016; Ben-David *et al.* 2017; Zdraljevic *et al.* 2017; Hahnel *et al.* 2018; O'Donnell *et al.* 2018; Brady *et al.* 2019; Zdraljevic *et al.* 2019). Over the last decade, molecular phenotypes such as transcript levels, protein levels, and metabolites have also been used in QTL mapping (Li *et al.* 2010; Vinuela *et al.* 2010; Singh *et al.* 2016; Snoek *et al.* 2017; Sterken *et al.* 2017; Gao *et al.* 2018; Sterken *et al.* 2019). Heritable variation in these molecular phenotypes often plays a role in heritable phenotypic variation (Jimenez-Gomez *et al.* 2010; Schmid *et al.* 2015; Sterken *et al.* 2017). Mapping expression QTLs (eQTLs) can provide insight into the transcriptional architecture of complex traits and have been conducted in model species such as *Arabidopsis thaliana* and *C. elegans* as well as several other taxa (Li *et al.* 2006; Keurentjes *et al.* 2007; West *et al.* 2007; Li *et al.* 2010; Rockman *et al.* 2010; Vinuela *et al.* 2010; Snoek *et al.* 2012, 2017; Cubillos *et al.* 2014; Ranjan *et al.* 2016; Sterken *et al.* 2017, 2019; Hartanto *et al.* 2020).

Most eQTL studies have been done on populations of recombinant inbred lines (RILs) originating from a cross between two different parental genotypes (Li *et al.* 2006; Keurentjes *et al.* 2007; West *et al.* 2007; Li *et al.* 2010; Rockman *et al.* 2010; Vinuela *et al.* 2010; Snoek *et al.* 2012, 2017; Cubillos *et al.* 2014; Sterken *et al.* 2017, 2019; Hartanto *et al.* 2020). The inclusion of more than two parents can capture more genetic variation, increasing the number of detected QTLs, potentially allowing more precise mapping and therefore reducing the number of potential candidate causal genes to be verified (King *et al.* 2012). Such a strategy was first used for *Arabidopsis* by developing a Multiparent Advanced Generation Inter-Cross (MAGIC) lines population consisting of 527 RILs developed from 19 different parental accessions (Kover *et al.* 2009). Several other MAGIC populations have been developed since then for a range of species, including *C. elegans* (de Koning and McIntyre 2017; Noble *et al.* 2017; Snoek *et al.* 2019).

Recently multiparental RIL (mpRILs) populations have been developed in *C. elegans* (Noble *et al.* 2017; Snoek *et al.* 2019). These populations have been created using other strains than the most frequently used N2 strain and the Hawaiian CB4856 strain (Li *et al.* 2006; Doroszuk *et al.* 2009; Rockman and Kruglyak 2009; Li *et al.* 2010; Vinuela *et al.* 2010; Rodriguez *et al.* 2012; Vinuela *et al.* 2012; Snoek *et al.* 2013, 2014, 2014, 2017, 2020; Stastna *et al.* 2015; Sterken *et al.* 2015, 2017, 2019, 2021; Thompson *et al.* 2015; Kamkina *et al.* 2016; Nakad *et al.* 2016; Singh *et al.* 2016; Jovic *et al.* 2017; Jovic *et al.* 2019; Evans *et al.* 2021). In this study, we used the population of 200 mpRILs, derived from an advanced cross between four wild types: JU1511 and JU1941 isolated from Orsay (France) and JU1926 and JU1931 isolated from Santeuil (France) (kindly provided by MA Félix, Paris, France; Volkers *et al.* 2013; Snoek *et al.* 2019). In a previous study, the RNA-seq data of these mpRILs were used to obtain almost 9000 SNPs variable between the four parental genotypes and used to identify QTLs for life-history traits (Snoek *et al.* 2019). The RNA was sampled from the mpRILs grown under standardized conditions (24°C, OP50, 48 h after bleaching) and obtained from animals from two 6-cm dishes, with one RNA-seq replicate per mpRIL and two per parental isolate. To investigate the effect of multiple genetic backgrounds on gene expression, we used the RNA-seq data to associate gene expression levels to genetic variants present in the population. We compared the gene expression level differences between the parental wild isolates, calculated transgression, as well as heritability and mapped eQTLs. We identified six *trans*-bands (TBs), hotspots at which many *trans*-eQTLs colocate, which we further studied by gene ontology enrichment. Lastly, we compared the eQTLs found in this study to the eQTLs found in previous eQTL studies in *C. elegans* (Li *et al.* 2006, 2010; Rockman *et al.* 2010; Vinuela *et al.* 2010; Snoek *et al.* 2017; Sterken *et al.* 2017). Together these results present the first insights into the genetic architecture of gene expression in a *C. elegans* multiparental RIL population.

## Methods

### Nematode strains and culturing, RNA-sequencing, construction of the genetic map

The *C. elegans* strains and culturing condition, RNA-sequencing, and construction of the genetic map can be found in Snoek *et al.* (2019). In short, the mpRILs used were grown in five separate batches with two 6-cm dishes per strain (24°C, OP50, 48 h after bleaching; the plates were randomized within incubators) and per strain, the two samples were pooled for RNA isolation, with one RNA-seq replicate per mpRIL and two replicates per parental isolate (Supplementary Table S4). Collecting and freezing the samples for one batch took approximately 30 min. The genetic map and eQTL profiles can found on WormQTL2 (Li *et al.* 2009) (http://www.bioinformatics.nl/EleQTL; Snoek *et al.* 2020).

### SNP calling and gene expression levels

The paired-end reads were mapped against the N2 reference genome (WS220) using Tophat (Trapnell *et al.* 2009), allowing for four read mismatches, and a read edit distance of 4. SNPs were called using samtools (Li *et al.* 2009), mpileup with bcftools and vcfutils as described in Snoek *et al.* (2019). Expression levels were determined using the tuxedo pipeline, giving length normalized fragments per kilobase per million (fpkm) values (Trapnell *et al.* 2012). Transcripts were assembled from the mapped reads using cufflinks (Trapnell *et al.* 2012). Raw RNA-seq data can be found in the Sequence Read Archive (SRA; https://www.ncbi.nlm.nih.gov/sra) with ID PRJNA495983. Normalized read counts can be found on WormQTL2 (http://www.bioinformatics.nl/EleQTL; Snoek *et al.* 2020). Normalization was done after the selection of the consistently detected transcripts (see QTL mapping and FPR) by taking the fpkm per gene per million fpkm per sample.

### Heritability and transgression

Heritability of gene expression levels was calculated using the heritability package in “R.” A narrow-sense heritability was calculated using the function *marker_h2* (Kruijer *et al.* 2015). The required kinship matrix was calculated using the *emma.kinship* function from the EMMA package (Kang *et al.* 2008). To determine a per-gene significance, we used a permutation approach where we shuffled the expression levels per transcript. After 100 permutations, the 95th highest value was taken as the 0.05 false discovery rate (Speed *et al.* 2012; Kruijer *et al.* 2015; Gilmour 2019). Transgression was determined by counting the number of mpRILs with an expression level beyond the mean + 2 SD of the most extreme parental lines. SD was calculated on the within-line variation of the parental samples. False-positive rate (FPR) was determined by permutations, randomly assigning the parental labels to gene-expression values. The threshold for transgression was set at an arbitrary 50 mpRILs (25% of all lines; FPR = 0.08) beyond the most extreme parental line(s).

### eQTL mapping and FPR

For eQTL mapping, we first selected the genes with consistently detected transcripts, meaning those expressed with a mean log_2_ expression (fpkm) >−5, which resulted in a set of 12,029 genes with transcripts that were detected in all samples. eQTLs were mapped by a linear model using a single marker model explaining gene expression (as log_2_ ratio with the mean) by one SNP-marker at the time for the whole genome. FPR was determined by one round of permutations where for each transcript, the counts were randomly distributed over the RILs before eQTL mapping. The -log_10_(p) value when number of false positives divided by the number of true positives was <0.01 [-log_10_(p) > 5.35]. Transbands (or eQTL hotspots) were determined for those loci that harbor more than 100 eQTLs in a 1-Mbp window to both sides of the marker under consideration. Genome-wide eQTL significance profiles [-log10(p)] can be found on WormQTL2 (http://www.bioinformatics.nl/EleQTL; Snoek *et al.* 2020).

### Enrichment analysis and figures

**Figure 1:**
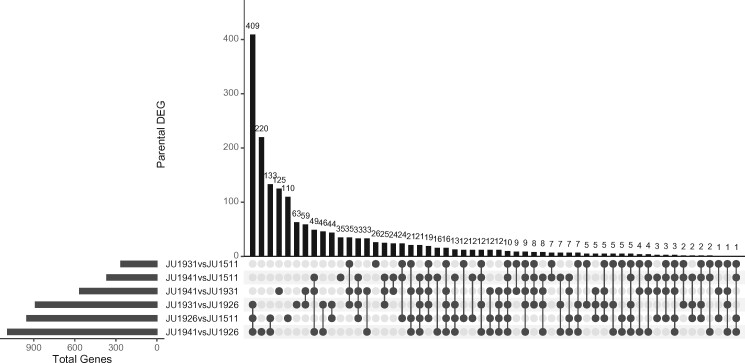
Gene expression differences between the four mpRIL parental lines. Upset plot shows the pairwise comparisons and the overlap between the pairs (Tukey’s HSD; *P* < 0.001; FPR = 0.05). The horizontal bar plot shows the number of DEG per parental pair, while the vertical bar plot indicates the number of shared DEG per comparison. For example, an overlap of 409 genes was found between the three comparisons that include the JU1926 parental line, which shows that JU1926 differed most from all other lines.

Enrichment of GO terms was done using the hypergeometric test in “R” (R Core Team 2017). GO term genes associations were download from Wormbase (http://www.wormbase.org) version WS276. Only genes that passed the filtering step for eQTL mapping were used as background genes. For significant enrichment, a *P*-value < 1e^−5^ was used and a geneset size per GO term >3. Most figures were made using the R package ggplot2 (Wickham 2009) except Figure 1 which was made using the UpSetR library.

### eQTL comparison between experiments/studies

To compare how many genes with an eQTL overlapped between the different studies (Li *et al.* 2006, 2010; Rockman *et al.* 2010; Vinuela *et al.* 2010; Snoek *et al.* 2017, 2020; Sterken *et al.* 2017) available in WormQTL2 (Snoek *et al.* 2020), we downloaded the eQTL profiles and markers used per experiment and listed the genes with a *cis-* or a *trans*-eQTL. For eQTL determination, the most significant marker per gene was taken as the peak. A -log_10_(p) > 3.5 was used as threshold for calling the eQTL. An eQTL was determined *cis* when the peak position was within 1 Mbp of the start position of the gene. These lists were compared with the genes having an eQTL in this study. The percentage overlap was calculated against the original study.

## Results

### Gene expression differences between the parental lines

To study the effect of genetic variation on gene expression, we used RNA-seq on a population of 200 multiparental recombinant inbred lines (mpRILs) (Snoek *et al.* 2019), made from a cross between four parental lines isolated from Orsay, France (JU1511, JU1941) and Santeuil, France (JU1926, JU1931) (Volkers *et al.* 2013). The animals used were grown on two 6-cm dishes (24°C, OP50, 48 h after bleaching) per sample pooled for RNA isolation, with one RNA-seq replicate per mpRIL and two per parental isolate. First, we determined the expression differences between the parental lines (Supplementary Table S1). Of the 12,029 detected transcripts, we found 1789 genes differently expressed between at least one parental pair (TukeyHSD *P* < 0.001; FPR < 0.05; Figure 1). Of the four strains, JU1926 was most different when compared to the other lines, with 409 genes being differently expressed between JU1926 and the other three lines. Thereafter, JU1941 was most different from the remaining two lines. These differences in gene expression between the parental lines are likely genotype dependent. To illustrate the reproducibility of the parental lines, we calculated the correlation between the parental samples and found that replicate pairs are formed. The correlation between the parental pairs is JU1511: 0.91, JU1926: 0.91, JU1931: 0.94, and JU1941: 0.82.

### Transgression and heritability

**Figure 2:**
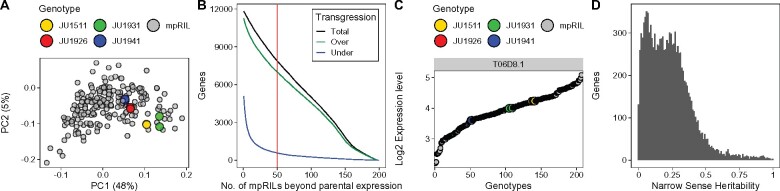
Gene expression variation in the mpRILs and parental genotypes. (A) Principal component analysis (PCoA) of the log_2_ ratios, mpRILs shown in gray, parental lines shown in color. (B) Transgression: number of mpRILs beyond the parental expression level (*x*-axis) against the number of genes (*y*-axis). The mpRILs below (under) the lowest parental expression level in blue, mpRILs over the highest parental expression level in green, and the sum of these (total) in black. (C) Example of two-sided transgression for expression levels of gene T06D8.1. (D) Genes with significant narrow-sense heritability (*h^2^*) and the distribution of heritable variation of gene expression variation at FPR = 0.05.

To explore the variation in gene expression between the different parental and mpRIL genotypes, we applied principal component analysis on the log_2_ gene expression ratios (Figure 2A). From exploration of the PCA axes, we concluded that there were no batch effects. This was also based on mapping (1) growth/sample batch, (2) RNA-isolation batch, and (3) sequencing batch. Neither of these traits mapped to the TBs that we detected. We can see that the expression variation in many of the mpRILs extends beyond the parental expression variation. The extension of variation suggests transgression and/or developmental variation. PC1 most likely corresponds to differences in development as gene families known to be strongly upregulated during L4 progression (Snoek *et al.* 2014) like vitellogenins (*vit*), major sperm proteins (*msp*), and chondroitin proteoglycans (*cpg*), were highly correlated with PC1. Analysis revealed transgression for 7896 genes (FPR = 0.08; Figure 2, B and C, Supplementary Table S2). Notably, most transgression was one-sided, showing increased expression level beyond the highest expression level found in the parental lines. This suggests that multiple segregated loci, in combination with developmental variation, are involved in regulating the transcription in the mpRILs. The mostly higher than the parental lines type one-sided transgression could also be caused by the developmental differences between the parental lines and the mpRILs, with most of the mpRILs showing a further developed expression profile. As a specific group of genes shows a large and progressing upregulation during L4, this could show up as one-sided transgression. Nevertheless, transgression often indicates that the trait variation, in this case gene expression levels, is heritable. We calculated the narrow-sense heritability (*h*^2^) and found significant *h^2^* for expression variation of 9500 genes (per-gene FPR = 0.05; Figure 2D, Supplementary Table S2). Most gene expression variation showed an *h^2^* below 0.5, indicating that part of the variation is caused by other factors than additive genetic effects. These other factors contributing to gene expression variation could be technical, environmental, but also more complex genetic interactions, such as epistasis.

### Expression QTLs

**Figure 3:**
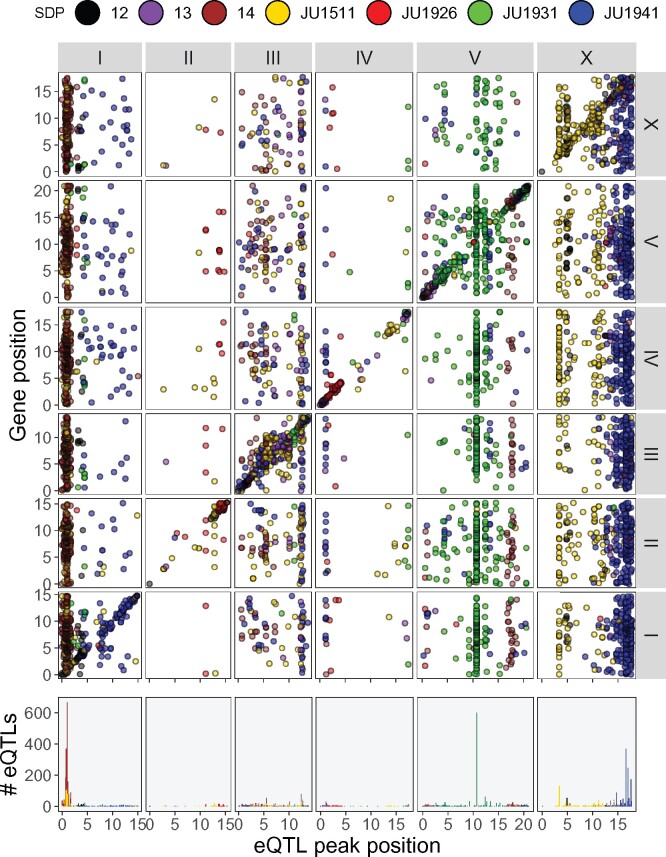
*Cis*/*trans* plot of the identified eQTLs. eQTL position shown on the *x*-axis, gene position shown on the *y*-axis (upper plot) or number of eQTLs (bottom plot). SDP shown in color, chromosomes shown in the gray strips on top and on the right of the panels.

**Figure 4:**
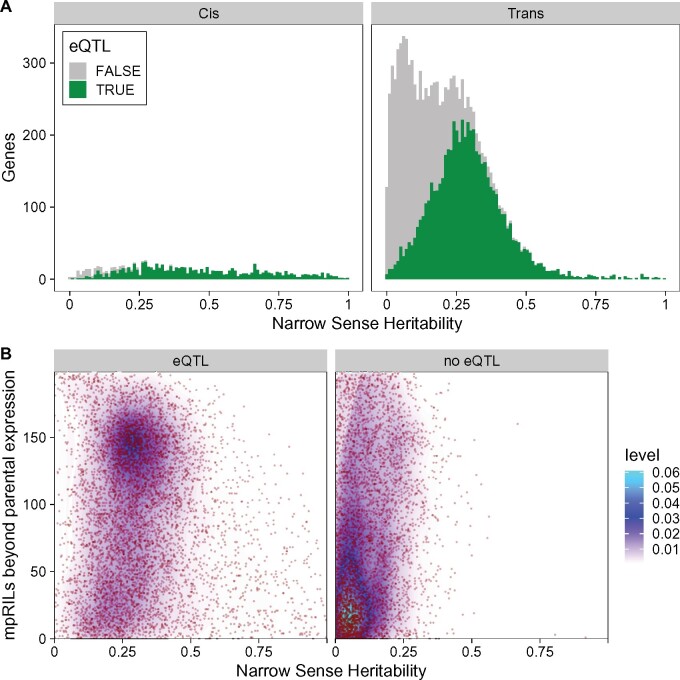
Relation between eQTLs, transgression, and narrow-sense heritability (*h^2^*). (A) Narrow-sense heritability (*h^2^*; *x*-axis), distribution in genes (*y*-axis) with *cis-* and *trans*-eQTLs, significance of the eQTLs is TRUE (green) when -log10p >5.35 and FALSE (grey) otherwise. (B**)** Relation between narrow-sense heritability (*h^2^*; *x*-axis) and transgression (*y*-axis) for genes with and without a significant eQTL, individual datapoints shown in red, color gradient indicates datapoint density.

To find the loci involved in gene expression variation between the mpRILs, we used a single marker QTL model. We found 6784 eQTLs (one eQTL per gene, -log_10_(p) > 5.35; FPR = 0.01), of which 929 were *cis-* and 5855 *trans*-eQTLs (Table 1; Figure 3; Supplementary Table S2). Most *cis*-eQTLs were found on chromosome V and most *trans*-eQTLs on chromosomes I and X. For both *cis*- and *trans*-eQTLs, fewest were found on chromosomes II and IV. The SNP Distribution Pattern (SDP) groups SNPs with the same distribution in the parental lines, for example the SNPs found in JU1511 and JU1941, but not in JU1926 and JU1931 share the same SDP. When the SDP is considered, many of the *cis*-eQTLs were found to have an effect where either the JU1511 or JU1941 allele was different from the three other parental genotypes. For the *trans*-eQTLs, the largest groups also show this allelic difference or those SNPs that distinguish JU1511/JU1941 from JU1926/JU1931. A substantial group of eQTLs was found for the JU1931 allele, whereas hardly any eQTLs were found for the JU1926 specific SNPs. The lack of JU1926 linked eQTLs is somewhat surprising as it had the most differentially expressed genes (DEG) in the comparison of the parental lines. Yet, we found much more genes with eQTLs than being DEG in the parental comparison. These are much more likely to be caused by new allelic combinations present in the mpRILs. Overall, the majority of the eQTLs are found on a few major effect loci with a specific SDP linkage (Figure 3). Moreover, comparing the *h^2^* to the eQTLs showed that genes with an eQTL have a much higher *h^2^* than those without an eQTL, where genes with an *h^2^* > 0.25 almost all have an eQTL (Figure 4). Comparing *cis*- and *trans-*eQTLs showed that genes with a *cis*-eQTL have a higher *h^2^* on average, yet the *h^2^* distributions of *cis*- and *trans*-eQTLs are overlapping.

**Table 1:** eQTLs per type (*cis*/*trans*) per chromosome per SDP

	*Cis*							*Trans*						
SDP	I	II	III	IV	V	X	Tot	I	II	III	IV	V	X	Tot
12 JU1511 and JU1926 *vs* JU1931 and JU1941	14	2	17	2	23	13	71	35	0	1	1	3	67	107
13 JU1511 and JU1931 *vs* JU1926 and JU1941	6	1	14	39	41	3	104	0	2	106	13	15	27	163
14 JU1511 and JU1941 *vs* JU1926 and JU1931	12	0	19	0	53	11	95	1373	0	119	5	103	44	1644
JU1511	37	32	61	14	18	81	243	457	28	211	20	9	430	1155
JU1926	0	32	4	59	5	1	101	5	44	5	26	10	5	95
JU1931	8	0	15	3	81	1	108	31	0	12	21	919	2	985
JU1941	76	0	66	5	38	22	207	150	1	155	35	94	1,271	1,706
Total	153	67	196	122	259	132	929	2,051	75	609	121	1,153	1,846	5,855

SDP, Chromosome, Peak position, and left and right borders in Mega-base pairs.

### Trans-bands

A large majority of the *trans*-eQTLs (3704; 63% of all *trans*-eQTLs) were found in six hotspots, so-called TBs (number of *trans*-eQTLs > 100, window 1 Mbp to both sides; Table 2; Figure 3). Two TBs were found on chromosome I, one on chromosome V, and three on chromosome X. The two TBs on chromosome I colocated but were linked to different SDP: the SDP 14 (JU1511/JU1941 *vs* JU1926/JU1931) and SDP JU1511 (*vs* the rest). The TB on chromosome V was linked to SDP JU1931 and the three TBs found on chromosome X were linked to SPD JU1511 and JU1941.

**Table 2:** Descriptive overview of the six identified TBs

	SDP	CHR	Peak (Mbp)	Left (Mbp)	Right (Mbp)	eQTLs	GO Enrichment (selection from enrichment table)	**Phenotypic QTL [**in Snoek *et al.* ( 2019)]
**TB1**	14 (JU1511 and JU1941 *vs* JU1926 and JU1931)	I	1.03	0.03	2.03	1,339	Thermosensory behavior, negative regulation of engulfment of apoptotic cell, DNA replication, embryonic body morphogenesis, establishment or maintenance of actin cytoskeleton polarity, muscle fiber development, epidermis development, response to unfolded protein and, molting cycle, collagen, and cuticulin-based cuticle	Population growth on *Erwinia* and on *B. thuringiensis*
**TB2**	JU1511	I	0.83	0	1.83	443	Regulation of protein stability, regulation of vulval development, DNA replication, anaphase-promoting complex and, microtubule polymerization	NA
**TB3**	JU1931	V	10.74	9.74	11.74	607	Hemidesmosome assembly, external side of plasma membrane and, negative regulation of response to oxidative stress	NA
**TB4**	JU1511	X	3.40	2.40	4.40	133	Adenyl-nucleotide exchange factor activity	Heat-shock sensitivity
**TB5**	JU1941	X	14.69	13.69	15.64	225	Dendrite morphogenesis	Population growth on *B. thuringiensis*
**TB6**	JU1941	X	16.60	15.65	17.6	957	Embryonic body morphogenesis, DNA replication, integral component of peroxisomal membrane, anaphase-promoting complex, endosome, phagocytic vesicle membrane, neuronal signal transduction, response to anoxia, cuticle pattern formation, cell fate commitment, hemidesmosome-associated protein complex, and response to lipid	Sensitivity to oxidative stress

Selection of enriched GO terms from Supplementary Table S3 and overlap with phenotypic QTLs found in Snoek *et al.* (2019).

### GO enrichment

To study the effect of TBs on the biological function, we used GO term enrichment (Table 2, Supplementary Table S3). Each of the TBs was linked to mostly different sets of GO terms, suggesting an effect on different parts of *C. elegans* biology. The genes mapping to TB1 on chromosome I were enriched for behavior and muscle and epidermis development GO categories. The genes mapping to TB2 on chromosome I were enriched for the GO term “vulval development,” among others. The genes with a *trans*-eQTL on TB3 on chromosome V were enriched for GO terms associated with oxidative stress. The genes mapping to TB4 and TB5 on chromosome X only showed a few enriched GO terms, among which adenyl-nucleotide exchange factor activity and dendrite morphogenesis. The genes mapping to TB6 on chromosome X were enriched for the GO term “response to anoxia” and many more. This shows that these TBs can be involved in several developmental processes and in the interaction with the environment.

### Overlap with other eQTL experiments

To investigate if the genes with eQTLs found in the present mpRIL study also had eQTLs in other studies, we compared them with the studies found in WormQTL2 (Table 3; Li *et al.* 2006, 2010; Rockman *et al.* 2010; Vinuela *et al.* 2010; Snoek *et al.* 2017, 2020; Sterken *et al.* 2017). In general, we found that a substantial group of genes with a *trans*-eQTL in any of the studies had an eQTL in our mpRIL experiment (26.5–36.9%). The groups of genes with *trans*-eQTLs show much higher overlap than the genes with a *cis*-eQTL in any of the experiments (10.2–20.0%). Around a third of the genes with a *trans*-eQTL in Vinuela *et al.* (2010), Snoek *et al.* (2010, 2017), and Sterken *et al.* (2017) also showed a *trans*-eQTL in the mpRILs, with numbers almost equal between developmental stages and treatments. Slightly fewer overlapping genes with eQTLs were found with Rockman *et al.* (2010) and Sterken *et al.* (2017). Comparing the experiments performed with the same N2 × CB4856 in the same lab (Li *et al.* 2006; Snoek *et al.* 2010, 2017; Vinuela *et al.* 2010; Sterken *et al.* 2017) shows that environmental conditions and developmental stage only have a small effect on the global overlap and difference between *cis*- and *trans*-eQTLs. As the genetic backgrounds of the mpRILs are different from the N2 × CB4856 populations used in the other experiments, the low percentage of overlapping *cis*-eQTLs could be expected. The large group of genes with a *trans*-eQTL in both experiments shows that the expression levels of a substantial group of genes are more prone to be affected by genetic variation independent of environment or developmental stage, while the loci involved are most likely different in each experiment/condition (Vinuela *et al.* 2010; Snoek *et al.* 2017; Sterken *et al.* 2017).

**Table 3:** Overlapping eQTLs between this mpRIL experiment and the RIL experiments available in WormQTL2 (Nijveen *et al.* 2017)

eQTL experiment	Total *Cis*	*Cis* Overlap (%)	Total *Trans*	*Trans* Overlap (%)
16°C (Li *et al.* 2006)	240	14.6	817	31.6
24°C (Li *et al.* 2006)	337	12.2	998	30.5
Li *et al.* (2010)	752	14.5	3,544	28.7
Rockman *et al.* (2010)	1,958	12.0	2,792	28.8
Control (Snoek *et al.* 2017; Sterken *et al.* 2017)	961	17.1	1,,481	36.1
Heat-shock (Snoek *et al.* 2017; Sterken *et al.* 2017)	976	20.0	2,776	36.9
Recovery (Snoek *et al.* 2017; Sterken *et al.* 2017)	992	16.1	1,519	33.4
Sterken *et al.* (2017)	719	10.2	1,116	26.5
Juvenile (Snoek *et al.* 2010; Vinuela *et al.* 2010)	303	11.9	2,206	33.4
Old (Snoek *et al.* 2010; Vinuela *et al.* 2010)	220	15.0	1,790	34.9
Reproductive (Snoek *et al.* 2010; Vinuela *et al.* 2010)	348	13.2	2,010	32.7

Percentages indicate the percentage of eQTLs found in the indicated experiment that are also found in the mpRILs eQTLs. Threshold used for the eQTL experiments in this table: -log10(p) >3.5; *Cis*-eQTLs were called if the peak of the eQTL was within 1 Mbp of the gene start, otherwise it was called a *trans*-eQTL.

## Discussion

In this experiment, we used a population of mpRILs and RNA-seq to find 6784 eQTLs, of which 929 were *cis*-eQTLs and 5855 were *trans*-eQTLs. A large proportion (63%) of the *trans*-eQTLs were found in six TBs. The total number of eQTLs found in this mpRIL study (6784) is at the high end of what was previously found in other experiments (mean: 2560; 653–6518) (Li *et al.* 2006; Rockman *et al.* 2010; Vinuela *et al.* 2010; Snoek *et al.* 2017; Sterken *et al.* 2017). This number is hard to compare as the number of identified eQTLs depend on many factors, such as population size, number of recombinations, statistical model, and RNA measurement technology used, which are nearly all different between this and the other eQTL studies in *C. elegans* (Li *et al.* 2006; Rockman *et al.* 2010; Vinuela *et al.* 2010; Snoek *et al.* 2017; Sterken *et al.* 2017). Nevertheless, it seems that a combination of RNA-seq and multiple genetic backgrounds increased the number of detected eQTLs. A very clear increase was found for *trans*-eQTLs (5855) compared to the numbers found in previous studies, even at a much lower significance threshold. For example, the study of Rockman *et al.* (2010) used a comparable number of recombinant inbred advanced intercross lines (RIAILs) as the number of mpRILs in this study (∼200), yet found fewer *trans*-eQTLs, however, the different conditions and technologies used prevent any definitive conclusions. With respect to *trans*-eQTLs, we do know that they depend on environmental conditions or a response to changing conditions. It could be that with a background of four parental genotypes the mpRILs perceive the ambient environment in a broader range than the RIAILs with a background of two parental genotypes used by Rockman *et al.* (2010), and the RILs in the other studies. For example, the mpRILs could have inherited parts of four different sets of environmental preferences as opposed to two in the RIAILs and RILs, potentially extending the accompanying gene expression patterns and eQTLs. Yet, the most likely reason for the increased number of *trans*-eQTLs is the use of RNA-seq in this study compared to microarrays in the other studies. Another reason for finding more *trans*-eQTLs could be due to the generally genome-wide equal allelic distributions in this population (Snoek *et al.* 2019). Namely, a similar TB as the chromosome I TB at 1 Mb (TB1) related to development has been spotted in other datasets, but has been spurious due to being located near the *peel-1 zeel-1* incompatibility locus, therefore, lacking recombinations in the N2 × CB 4856 RIL panel use before (Li *et al.* 2006; Seidel *et al.* 2008; Li *et al.* 2010; Snoek *et al.*  2017, 2020). Another advantage of using RNA-seq is that the genotype and gene-expression levels can be obtained from the same sample, preventing mislabeling errors and the need for “reGenotyping” in case of microarrays (Zych *et al.* 2017). In summary, as has been shown for yeast (Albert *et al.* 2018), the combination of generally smaller effect of *trans*-eQTLs and higher dynamic range of RNA-seq would at least increase the possibility to pick-up *trans*-eQTLs in *C. elegans* and in general.

It is noted that genetic variation in development across the mpRILs could be an important driver of *trans*-eQTL hotspots (Francesconi and Lehner 2014). We found two eQTL hotspots that are enriched in GO terms related to development. As the mpRILs differ in developmental speed, it would be interesting to include it as a covariate and assess its weight in the mapping as has been described in Francesconi and Lehner (2014). To allow for comparison with previous eQTL studies, we decided not to include development as cofactor in our analysis since this was also not done in the earlier studies (Rockman *et al.* 2010; Francesconi and Lehner 2014).

We previously found QTLs for several different phenotypes, such as population growth on different bacteria, sensitivity to heat shock and oxidative stress (Snoek *et al.* 2019). Four TBs were found to colocate with the previously found phenotypic QTLs (Table 2). Population growth on *Erwinia* and on *Bacillus* *thuringiensis* DSM was found to colocate with TB1, which was enriched for GO terms related to muscle, epidermis, and molting. This could indicate a difference in these structures that can affect the interaction with different types of bacteria or could indicate that there is a difference in developmental speed through which differences in the expression, and subsequent eQTLs, of molting-related genes are picked up. A QTL for heat-shock sensitivity was inferred to colocate with TB4, however, no indication for a link with this phenotype was found in the annotation of the genes with an eQTL at this position. The same was observed for TB5 and the overlap with population growth on *B. thuringiensis*, where GO enrichment also did not provide any leads to a potential mechanistic link. The overlap between the QTL for sensitivity to oxidative stress and TB6, however, did show some clues from GO enrichment as genes involved in the peroxisome as well as DNA replication and cuticle formation could be involved in dealing with oxidative stress.

We expect to have only found a fraction of the eQTLs, as we only used a simple additive mapping model, a conservative score of one eQTL per gene, and standard lab conditions with only one time point for RNA isolation. Both the number of eQTLs and genes with one or more eQTLs are expected to increase when more complex models are applied to these data and/or different experimental conditions and time points are considered (Vinuela *et al.* 2010; Francesconi and Lehner 2014; Snoek *et al.* 2017). Moreover, we use an SNP-based method for eQTL mapping, which has a binary option for each marker and therefore does not consider the genetic origin (parent) of the SNP. Using the genetic origin of the SNPs could reveal the more complex genetic interactions that could underlie the differences in transcript levels between the mpRILs. These complex genetic interactions are suggested to be present in this mpRIL population, by the heritability and transgression found. A model in which each marker has the four parental options might indicate loci with more than two alleles affecting gene expression. Furthermore, some (relatively small) genetic loci might have been missed all together as our investigations are based on the N2 reference genome and wild isolates can have vastly divergent regions of which sequences reads fail to align to the N2 reference genome with conventional methods (Thompson *et al.* 2015; Lee *et al.* 2021).

This study provides a more detailed insight into the genetic architecture of heritable gene expression variation in a multiparent recombinant inbred population. The use of RNA-seq data in combination with more than two alleles allows for a more precise detection of QTLs and incorporates a wider band of standing genetic variation, resulting in a substantial increase in eQTLs especially *trans*-eQTLs. Comparison to bi-allelic studies supports the position of eQTLs and may be used to detect a more detailed pattern of associated loci. We expect this study, data, and results to provide new insights into *C. elegans* genetics and eQTLs in general as well as to be a starting point to further test and develop advanced statistical models for detection of eQTLs and systems genetics studying the genotype–phenotype relationship.

## Data availability

The data underlying this article available in Sequence Read Archive (SRA; https://www.ncbi.nlm.nih.gov/sra) and can be accessed with ID PRJNA495983 and in WormQTL2 (http://www.bioinformatics.nl/EleQTL; Snoek et al. 2020).

Supplementary material is available at *G3* online.

## Supplementary Material

jkab258_Supplementary_Data
